# Through the eyes of community health workers: what was needed to increase COVID-19 vaccine uptake in the Missouri Southeast region

**DOI:** 10.3389/fpubh.2024.1286177

**Published:** 2024-03-27

**Authors:** Anusha Ban, Ashish Shrestha, Carissa Van den Berk-Clark, Janice Ballard, Richard Logan, Tripp Logan, Anne Francioni, Megan Murray, Elizabeth A. Baker

**Affiliations:** ^1^College for Public Health and Social Justice, Saint Louis University, Saint Louis, MO, United States; ^2^Department of Family & Community Medicine, Saint Louis University, Saint Louis, MO, United States; ^3^One Heart Many Hands, Caruthersville, MO, United States; ^4^L and S Pharmacy, Charleston, MO, United States; ^5^Whole Kids Outreach, Ellington, MO, United States; ^6^Southeastern Missouri Area Health Education Center, Poplar Bluff, MO, United States

**Keywords:** community health workers, rural health, COVID-19, vaccine equity, community engagement

## Abstract

Public health emergencies, such as the COVID-19 pandemic, elucidate the strengths, weaknesses, and significant gaps in infrastructure, compatibility and consistency in communication systems, as well as the quality of collaborative relationships, and provider and workforce capacity. They also expose longstanding patterns of mistrust in the government and healthcare systems, and inadequacy in socio-economic infrastructures. These issues resulted in higher COVID-19 infection and mortality rates, and lower vaccination rates in many rural counties across the nation, including Missouri. In response to these challenges, the COVID-19 Response Network was formed in the Southeast corner of the state. The Network was a community-academic partnership that brought together community and faith-based leaders, academicians, healthcare providers and administrators, public health practitioners, and pharmacists to facilitate collaboration on education and outreach efforts aimed at reducing vaccine inequity in the 16-county project area. Importantly, the Network also included Community Health Workers (CHWs) who worked with these different agencies and organizations and were at the heart of implementing Network activities. The intent of this study was to assess their perspectives on the factors that influenced community engagement and communication strategies, and increased vaccine uptake in rural Missouri. Qualitative methods, including in-depth interviews, were used to explore the professional and personal experiences of CHWs working at the grassroots level during an ongoing pandemic. Narrative analysis revealed effective communication and engagement strategies for increasing vaccine uptake in rural communities. For instance, fear-based messaging was perceived as coercive and met with resistance. In contrast, messages that shared personal experiences and catered to the human need to protect their loved ones were more effective. Trust in the source of information was critical. This study highlights the significance of exploring and leveraging the capacities of trusted community members like CHWs to increase the effectiveness of public health interventions in rural communities.

## Introduction

The Coronavirus Disease 2019 (COVID-19) presented unprecedented challenges to the State of Missouri, the US, and the entire world. Within rural counties of Missouri, including the study site, COVID-19 highlighted what we already knew about deficiencies in clinical, public health, and economic infrastructures, and magnified existing racial and economic inequities and distrust in the government and the healthcare system. COVID-19 safety measures such as wearing masks, testing, social distancing, and vaccination were controversial and politicized across these rural counties (1). Given these circumstances, rural Missouri experienced a higher COVID-19 mortality rate per 100,000 population (94.2) compared to both statewide (87.3) and urban rates (83.2) (2).

It is within the above context that the COVID-19 Response Network was formed to serve a 16-county rural area in the Southeast region of Missouri including Butler, Cape Girardeau, Carter, Dunklin, Howell, Iron, Madison, Mississippi, New Madrid, Oregon, Pemiscot, Ripley, Scott, Shannon, Stoddard, and Wayne counties. These counties have relatively low levels of racial and ethnic diversity. For example, while around 27.5% of Pemiscot County is comprised of African American persons other counties such as Carter, Ripley, Oregon have less than 0.5% of their population self-identifying as African American (3). Similarly, there are few individuals who self-identify as Hispanic/Latinx communities living in this 16-county area (Dunklin County being the highest at 7.2%) (3). Despite this, Black/African American persons living in rural areas were 80% more likely to die from COVID-19 than White persons living in rural areas (1).

With higher rates of poverty (15.9%) compared to the urban areas of the state (10.9%) (4) these rural counties rank very high or high on the Centers for Disease Control and Prevention (CDC)‘s Social Vulnerability Index (SVI) (Mean SVI = 0.69 ± 0.26) as well. This measure includes socioeconomic, population, and housing/transportation vulnerability measures with scores ranging from 0 representing lowest vulnerability to 1 representing highest vulnerability (5). These counties have also been designated as health professional shortage areas. For example, the range of primary care providers per 100,000 population in these southeast counties of the state range from 0 providers per 100,000 population in counties like Shannon and Oregon to 90–100 providers per 100,000 population in counties like Cape Girardeau and Butler (6). Having fewer primary care providers *per capita* has been linked to lower life expectancies (7). Furthermore, the county rankings and roadmaps indicate that these rural counties also experience some of the worst health outcomes and behaviors in the state (8).

During the pandemic additional measures of vulnerability were also utilized, for example the COVID-19Vaccine Coverage Index (CVAC) measured how well a community could handle the repercussions of a COVID-19 outbreak and was based on a community’s access to health care, affordable housing, transportation, childcare, and safe and secure employment (9). The CVAC Index values range from 0 (least vulnerable) to 1 (most vulnerable) and it is categorized as follows: Very Low (0.0–0.19), Low (0.20–0.39); Moderate (0.40–0.59); High (0.60–0.79); and Very High (0.80–1.0). Most counties in these rural counties of Missouri were found to have high or very high vulnerability to handle a COVID-19 outbreak (Minimum: Maximum: Mean CVAC = 0.64 ± 0.06) (9).

### Establishing a community-academic network

It is within this context that the COVID-19 Response Network was developed. It brought together community and faith-based leaders, primary and mental healthcare providers, pharmacists, academic institutions, and CHWs affiliated with different community - based agencies. The Network met virtually (via Zoom) weekly prior to and throughout the project period (March 2020 through August 2022). During these meetings, community, and academic facilitators shared data on COVID-19 for the region, the state of Missouri, and the US, as well as resources available (financial, material and informational) in and beyond the state to mitigate the challenges raised by the pandemic. The meeting participants contributed by sharing resources available through their organizations and in the community with other participants along with information about COVID-19 related events and locations where education, awareness, and vaccination activities would be performed. These joint efforts also led to a number of diverse COVID-19 Response Network actions including mobile food banks; the creation and distribution of health infographics tailored specifically for the community; and the organization of vaccination events in collaboration with local community pharmacies (8).

### Purpose of the study

The intent of this study was to assess community health worker perspectives on the factors that influenced community engagement and communication strategies, and increased vaccine uptake in rural MO. This paper focuses on the experiences and perspectives of CHWs regarding community engagement during the COVID-19 pandemic, specifically documenting the communication strategies that enabled them to be successful at providing education regarding COVID-19 and encouraging vaccine uptake in the rural counties of Missouri. The study aims to further provide insights into the potential of CHWs to contribute to the health-seeking behaviors of individuals living in rural areas of the US.

## Methods

### Study population

A total of 65 CHWs from three different partner organizations were mobilized to conduct community outreach events in multiple counties within the Southeast region of Missouri. A little over half of the CHWs (55%) were employed by the partnering pharmacies, and the other half were employed by community-based organizations. Most of these CHWs (89.2%) were females and 20% were between the ages of 18–25, while around 77% were over the age of 25. Efforts were made to hire CHWs that represented the most vulnerable populations in our 16-county area. Sixty-nine percent of our CHWs lived in the same community or in one of the communities where they worked. Majority of the CHWs identified themselves as White (77%), while 17% of them identified themselves as Black or African American. This racial breakdown suggests an overrepresentation of African American given the demographic background of our study area. Almost all (95%) CHWs involved had completed a GED or a high school diploma. Additionally, 43 % of the CHWs reported having worked prior to the pandemic as CHWs in various capacities such as outreach coordinator, community liaison, healthcare and social services navigator, health educator, as well as community health worker.

Of the total sample of 65 CHWs, a sub sample of 11 were recruited through stratified (by organization that employed them) convenience sampling to participate in a semi-structured qualitative interview. Their demographic characteristics mirrored that of the 65 CHWs in general (e.g., 77% white, 90% female). Similarly, among the eleven study participants, eight of them self-identified as white and the remaining as African American.

### Study design

This observational study used designed to capture the unique reflections of CHWs while conducting community-based educational outreach events to improve the uptake of COVID-19 vaccines within the 16-county area of Missouri. Hence, it employed a narrative qualitative approach to understand both the personal and professional insights gained through the efforts made to promote vaccination uptake. Quantitative data were used to capture CHW demographics, the number of educational events, and vaccines delivered.

### Data collection and analysis

Members of the community and academic members of the research team reached out to CHWs via email, and provided an information statement about the study, requesting their participation. The interview protocol focused on the context within which CHWs conducted their work, community perceptions about COVID-19 and the vaccine, strategies and messages used for educational outreach, and CHW impact on a community as well as systemic level. Interviews were conducted and recorded via zoom after receiving the verbal consent of the participant. Transcription was completed using Zoom application’s written transcription option, and these written transcripts were compared to audio recordings by the team’s research assistants to ensure accuracy. All 11 transcripts were then analyzed using an inductive approach, beginning with open coding followed by a focused coding technique in which multiple members of the research team independently assigned codes to transcripts based on the interview topics, discussed disagreements, and came to a consensus on the best way to address discrepancies to ensuring intercoder reliability. After initial coding, codes were then arranged into thematic clusters with summary paragraphs synthesizing key elements from each thematic cluster, along with supporting quotations from the interviews.

Through shared online forms (Qualtrics), partner organizations collected quantitative data to identify CHW demographics, the number of educational events, and vaccines delivered.

## Results

Through the interviews, CHWs provided an insight into the context within which they worked. They shared experiences related to the different strategies employed to navigate through that context and reach community people where they are. These experiences and insights have been documented in the CHW’s original words and have been presented below in four major thematic clusters: context, content, strategies, and trust.

### Context within which the CHWs conducted their work

CHWs stated that most of the community members were not initially concerned about the pandemic as they had ‘bigger issues’ they needed to focus on, and many individuals in the community did not believe that COVID-19 was “real” (1a, 1b). CHWs also noted that initially when the vaccine came out, many people were ‘anxious and scared’ about getting the vaccine, and some families and community members had their ‘mind set against’ it (1c, 1d).

According to the CHWs, they experienced some pushback from the community members during community events, which they believed might have been due to misinformation about COVID-19 and the vaccine (1e). Furthermore, they noted that differences in political ideologies influenced community perspectives about the pandemic and the vaccines (1f). They indicated that social media platforms (including Facebook, Instagram, and TikTok) was their ‘worst enemy’ and the ‘biggest backset’ exacerbating much of the misinformation and polarization (1g).

CHWs also noted that lack of social support for getting vaccinated, or social norms against vaccination, made it difficult for some community members to get vaccinated (1h, 1i). Moreover, CHWs stated that families often created what could be called a “cultural block” within the communities which made it difficult for families to receive and accept information (1j). Some CHWs also raised similar concerns about the impact of the medical and social service organizations on community members’ decisions to get the COVID-19 vaccines. They noted that some medical professionals in these rural communities told their patients that they were against vaccinations (1k).

### Content: messaging for rural communities

Given this context, CHWs noted that certain messages were particularly effective in increasing vaccine uptake among community members. For example, CHWs spoke about how sharing their own personal values and experiences with COVID-19 and the vaccines helped to shift community members’ mindsets, encouraging them to receive vaccines against COVID-19 (2a, 2b).

CHWs also indicated that family members, peers and people in their lives played a critical role in motivating their decision to vaccinate themselves (2c); the key driver being the desire to protect vulnerable family members. CHWs stated that recounting these personal stories about the process of accepting the vaccines at outreach events as a messaging strategy resonated with community members and helped them to understand the importance of the vaccine and even change their perspectives towards vaccines (2d, 2e). Sharing clear and easy to understand statistics about hospitalization and loss of life due to COVID-19 also played a critical role in motivating community members to obtain vaccines (2f, 2 g).

In addition, working in a diverse community setting with differing levels of understanding required CHWs to tailor the information provided in deference to the individuals or groups they interacted with (2h). For instance, catering to people’s beliefs or faith, was helpful in getting through to some groups (2i).

### Strategies for engaging rural communities

#### Using neutral, non-coercive strategies and handing over the power to decide

CHWs indicated that their success was not just due to what they said to community members, but how they said it. They recognized the importance of being mindful of how the information is provided, starting from the moment they approached an individual (3.1a). They emphasized the importance of framing messages in ways that were appropriate for their communities. CHWs also noted that the level of credibility of the individual delivering the information was seen as equally important (3.1b) Apart from credibility, CHWs emphasized that treating people with respect, being polite, and greeting them with a welcoming attitude made community individuals receptive to the information provided (3.1c). Using appropriate, non-coercive words (3.1d, 3.1e) to offer information on COVID-19 and the vaccine against it was effective in creating an open dialog that people could return to. CHWs who sought to provide community members with options and a sense of control over their own decisions (3.1f, 3.1g) were met with less resistance (3.1 h). Furthermore, taking a “neutral standpoint” (3.1i) when providing necessary educational information on COVID-19 and vaccines helped CHWs address misinformation (3.1j) and enabled community members to make informed choices.

“Meeting people where they are” (literally and figuratively) and being polite often helped CHWs to develop trust and rapport with community members (3.1k). In their experience, it was equally important to use easy to understand plain language (3.1l) and visually appealing educational aids such as flyers and audiovisual aids (3.1m, 3.1n). Whatever the mix of strategies for engagement, they also highlighted that to be effective they needed to interact with people in ways that left the door open, allowing for follow up conversations (3.1o, 3.1p). Using educational materials to facilitate a two-way conversation as opposed to a one-way narration of information was met with more ‘acceptance’ by the community members (3.1q).

#### Using insistent, forceful language, fear-based messaging was ineffective

Trying to force families to get vaccinated was seen as ineffective and in certain cases worsened existing doubts and fears people had about vaccinations. One example where insistent language was used, the individual decided not to get vaccinated, and this also had a detrimental ‘domino effect’ resulting in them discouraging vaccinations for their children as well (3.2a).

Instances where fear-based messaging was used or when the CHWs just told people to get vaccinated without providing educational information reportedly created increased resistance (3.2b). While most CHWs found using different forms of Supplementary educational materials helpful (3.2c), some noted that many community members also preferred to engage in conversations rather than reading information from a piece of paper or having someone read information to them but was important to have it available (3.2d).

#### Strength in partnership

CHWs shared the positive changes brought about through the outreach efforts conducted in collaboration with community pharmacists and other community-based organizations. They noted that along with the efforts to promote preventive strategies against COVID-19 CHWs also responded to any needs or challenges presented during the pandemic as a ‘united front’ (3.3c). Particularly the collaborative relationship established with pharmacists was quoted to be a ‘big change’ which did not exist prior to the formation of the Network (3.3b) and continued to last beyond the completion of the project (3.3a). Apart from this, partnership with other organizations led to increased accessibility to more resources and provided CHWs with the ability to refer community members to relevant resources and services when necessary (3.3d).

#### Meeting community members where they are

CHWs relied heavily on partnerships with community organizations to increase their capacity to intervene in person. One of the most used approaches was to be a part of existing community events. Conducting outreach events at locations frequented by community members enabled CHWs to reach people “where they are.” CHWs also organized their own social events such as ‘luncheons’, ‘pageants’ etc. that offered a combination of entertainment, information, and service (opportunity to vaccinate) that not only ‘attracted’ people but was also a successful engagement strategy for encouraging vaccine uptake (3.4a, 3.4b). CHWs also noted that apart from the content and its delivery, it was necessary to be mindful of ‘who’ was present at the event, and who was providing the information at the event. Having pharmacists provide technical information within the context of such events further increased the positive impact of the information provided (3.4c). This was even more profound when CHWs went into communities they were not familiar with, and the trust factor had not yet been established (3.4d). A big part of meeting community members where they are, and the community outreach efforts conducted by the CHWs was the ability to have ‘face-to-face’ conversations which were noted to be a more effective medium of communication compared to phone calls (3.4d, 3.4e).

### Trust was the key factor

In addition to having the right message and medium, when asked about how the community received them, CHWs felt that a key to their success was that the community trusted them regarding the information they provided on COVID-19 and the vaccines, which helped community members make their own decision. A major factor contributing to the community’s trust in the CHWs was related to them already being known and ‘embedded’ within the community (4c). As a result, community members felt ‘comfortable’ expressing their views to these CHWs (4a, 4b, 4d). Importantly, as noted earlier, the majority (69%) of the CHWs lived in the same community where they were working as a CHW for this project. The trust factor extended beyond CHWs to the broader community, as observing fellow community members getting vaccinated or learning about their vaccination decisions through social media played a significant role in influencing individuals’ own decisions regarding vaccination (4e, 4f) (Tables 1–4).

**Table 1:** Community context.

“They (community people) were not negative towards me (CHW) or the information, but like they have bigger issues and things that they needed to focus on, so, and they already had their minds made up that they were not going, they were not worried about it (the pandemic) or that they did not need any information”
“There were people that did not even believe that COVID was real whenever I (CHW) was first going into their home”
“They (community people) were scared at first…”
“I’ll (CHW) just think there were people in the community that had their mind set, and no matter how much telling you did to them, they were not going to listen (to get vaccines)”
“I’m (community individual) not ‘gonna do it (get the vaccine) because a magnet would stick to my head, we (CHW) heard that stuff like that a lot. And that the government was putting poison in them (vaccines)”
“It’s (vaccination) the government’s way of controlling you, and nobody’s gonna make me (community people) get this”
“I would say the media was like our worst enemy because everyone goes on Facebook, social media and all of that stuff for their information. And, as you know, not all of that’s 100% factual… And so that was honestly, the biggest backset was social media, and what others were saying about it (vaccines)”
“it’s really hard to get them (community people) to open up, because in order for them to open up, they have to also kind of face their family, or their loved ones, that have different opinions. And being so secluded here, whenever you are, you know when you have such a differing opinion with the 5 people that you are close with, that feels really isolating more than they already are. So, it is kind of hard to get them to accept new information”
“I (CHW) think that a lot of it (vaccination) was that you know these, the people in the community, even if they are interested in getting vaccinated, or they do want more information, they are hearing so much negative feedback from their family members at home that they might be afraid to reach out and do what they feel is right because of the judgment they would receive from the people that are close to them”
“There’s just a little bit of a culture block here (in the community). It was just really hard for the families in this area to receive this information and be very accepting of it”
“The craziest thing is the worst people with this (perspective of medical community towards vaccine) is the people that’s in the nursing school. The nursing classes. The nursing class was the worst class of students that was against the vaccination”

**Table 2:** Messaging for rural communities.

“Basically, I (CHW) will let them (community people) know that I personally took the vaccination, and I did not have any complications or anything. So really just letting them know what they want to know “how I’m gonna feel afterwards, like we have a sore arm…,” you know what you are gonna be fine.”
“A lot of Community, they (community people) already knew me and a lot of people already knew my stance on the vaccine, they know that I wasn’t kind of pro vaccine and then, when I was going back and telling them that I ended up taking the vaccine, they asked me what changed, what made you do that, and that’s when I was able to really inform them or give them more information and give them reasons why I made the change”
“My (CHW) God daughter… She’s a travel nurse and she was working in the COVID Ward, and she saw so many deaths out there so many people died… she was adamant that I get vaccinated because of what she saw. The experiences she had, and so that’s what made me have a transitional thought pattern in my mind and then I decided to go and get vaccinated.”
“I (CHW) said, ‘well in all honesty, I would do it (take the vaccine), especially if you have grandparents or parents, (older adult) people’. If they have any kind of chronic disease, it’s really worth taking the vaccination… you have diabetes or you have a grandma, grandpa or older (relative)… your mom or dad. You do not want to catch it, and then they have these chronic diseases, and that could coincide with that health issue, it can lead to death”
“My (CHW) main reason for getting this shot… it’s not so much about me, even though it scares me to think I could be laying there on a ventilator… it’s for my family, it’s for my grandchildren… I said that I need to protect her, and I need to protect your children, and that’s how I viewed it. It wasn’t about me and who was forcing something on me. It was me helping protect the people I loved. I turned it around (the messaging), and sometimes they are (community individuals) like, oh, okay! they could kind of see it a little differently.”
“… and then 1 week three city leaders, 50 years of age and younger died. We had three people die in 1 week, and they were prominent citizens. One was our prosecutor. Another was a highway patrolman and the other worked for the city as a dispatcher. That was really sobering. So, after those unfortunate tragedies we had more of an outpouring of people thinking `I think I’ll go ahead and get the vaccination anyway’”
“We did have some people that would change their mind and come in, say, for example, a couple of people I called, they said, well, I was not going to do it (get vaccinated), but my husband died…”
“Everybody is someone different, and so you (CHW) have to change who you are, what you say and how you say it, depending on who they (community people) are”
“I (CHW) want them (community people) to feel comfortable when they can talk to me about anything, and like I say, I’m just a Faith believer and that’s strong right there because some people - I’ve had people come up to me, and, say, ‘wow something about you, I do not know what it is. I would say it is only God, I give God all the credit. And he (God) just felt like I will be really good… helping the people and, so far, I mean it (using faith-based strategy) has worked”

**Table 3:** Strategies.

3.1 Outreach strategies for engaging rural communities (Neutral, non-coercive, power to decide)
“I (CHW) would open it (community events) up and tell them, like you know, “the extended family is welcome to come too. I’m going to be there tomorrow, I thought maybe we could talk about COVID and the vaccinations. All opinions are welcome, I’m here to answer any questions that you have. So, if your dad or your grandparents, or your brother would like to be there too, if they have questions, I can answer for them too…””
“Whenever I’m (CHW) giving them (community individual) the information verbally, like we are having a conversation, and I’m starting to say like statistics, you know, even if they receive it all well, I always leave the paper copy with them so that they can trace back trace back the information and say oh, this was actually legitimate, she wasn’t just making numbers up”
“Because I (CHW) always walk up to people with a smile and always you know to greet them with a smile, and I tried to do that and not try to push this on them
“What we (CHW) did not do, we did not get out in the field to try to persuade people, we did not get out to try to force it (vaccine) on them. The only thing that we were doing was getting out, trying to give them the right information, and at the end of the day, it’s up to you. What you want to do is kind of like your own decision. You want to go to heaven or hell? (Metaphor representing decision to vaccinate) Everybody has a choice on what they want”
“…the more you (CHW) say it (talk about vaccine), the more you do not want to feel like you (CHW) are just trying to push something down somebody’s throat, to make somebody feel guilty for not taking it or trying to force someone to get the vaccination”
“And so, without really, coming on too strong (during community outreach) without really pushing it (vaccine) on, then bring a little education here and there, and just kind of let them know that I (CHW) could answer any questions if they had. They did ask some, and I felt like they did accept the information that I was giving them”
“For people to be able to voice their thoughts, their concerns, their opinions, and to be able to have that freedom. to be able to engage, but you know, give them food for thought, but at the same time, let it be their decision to embrace what’s right for them and their family”
“…And so that was my approach with people. I did not really go to them and try to say you need to get your shot; you need to do this. No, I never did that, because I would not want anybody to do it to me, and so I just treated people the way I wanted to be treated and approached them in the same way I will want to be approached, and so I did not really have a lot of kickback…”
“We (CHWs) kind of just tried to take a neutral enough standpoint on it (vaccines) to just let them come to us for information, education, and feel comfortable. We had also offered confidentiality”
“I (CHW) had to help inform them that the vaccination itself - it will not stop me from getting COVID, it’ll help save your life…Some will say, “Well, I know this person got the shot and he still got COVID,” I say “Well it’s not preventing you from getting COVID, it’s trying to prevent you (from developing severe disease) if you got COVID it will not be as bad (severe) on you”
“You (as a CHW) have to remember, you have to meet people where they are. And you have to remember who you are, and not to say anything that’s going to be offensive, you know even, no matter how offensive, a person could be coming to you, you have to remember just who you are”
“She (CHW) uses these big words that nobody knows what she is talking about. She stood there for 15 min. And then I’m standing right beside her, basically breaking down everything she just said”
“We had made up flyers and pamphlets, so that way we could say we have done our research. This is what we found, and we want you to look it over at your own time, your own convenience, and I think that worked better because it was giving them the option to look at it or to not look at it”
“We (CHWs) also have videos that we could show them (community members), we have got laptops and tablets, that worked on a couple, they were pretty receptive to that”
“Make a follow up call about a month later, and just say, ‘hey, just wanted to check on you to see if you have any more questions about that kind of thing.’ and we did have some people that would change their mind”
“Tried to provide follow up and chances to ask or answer more questions”
“I’ve (CHW) noticed a trend with this area… a lot of these people do not like to feel like they are being taught. They do not mind sitting down and just have a conversation with you, but whenever you bring out papers and you are like giving it to them, or you are reading things off to them, or you are showing them an informational video more often than not… they just seem standoffish, like they are just really not accepting of the information”

3.2 Outreach strategies for engaging rural communities (insistent, forceful language, fear-based messaging)
“I’m (CHW) trying twor..k with moms in families for child checkups and regular immunizations, and that (when forcing them to get vaccines) had a domino effect. They went from, ‘you are not telling me to get the COVID shot’, to suddenly, ‘I’m not getting any of my kid’s shots’. I was like, you have gotta be kidding me. We just went back like 15 years!”
“You (CHW) cannot make people be afraid, and say, you know if you do not get this, you are gonna be sick. You cannot come at them like that, and so it’s about how you are talking”
“Coming out, and just talking about it without any backup like without any pamphlets or anything definitely did not work whatsoever”
“I’ve (CHW) noticed a trend with this area… a lot of these people do not like to feel like they are being taught. They do not mind sitting down and just have a conversation with you, but whenever you bring out papers and you are like giving it to them, or you are reading things off to them, or you are showing them an informational video more often than not… they just seem standoffish, like they are just really not accepting of the information”

3.3 Strength in partnership
“I (CHW) know just looking back on things, I met a lot of different pharmacists, and they are still open to, even though the pharmacy network kind of diminished at the end of last year, I still have pharmacists reaching out saying, “hey, if you still know anybody, send them over here,” you know, so I’m thankful for that little bit of change”
“So, I (CHW) think that’s for me, that’s a big change. Before this project, nobody, no pharmacist would call me and say hey do you have any name, so you have anybody, you know that is still wanting the vaccination, so I think that was a change as far as me personally, reaching out.”
“I (CHW) feel like, probably in the whole… community of pharmacies, the Health Center, the Ozark Action Agency – I feel like all of us together, it was like a united front. We all worked together, but we all PULLED together to try to get done what needed (to be) done. So, I felt like there was this…a closeness probably that was formed because we were all in this together that maybe wasn’t there… before that hopefully will stay, will continue with anything that comes up as a challenge in a community that we can face now that we have kind of you know…”
“And just being able to also have access to more resources, you know and referrals, we can refer more to other (organizations as a result of the connections we have made through this project)”

3.4 Meeting community members where they are
“We (CHW) offer door prizes in some of the churches to get the people to come. We had a luncheon, free lunch. Yeah. We gave them pizza, we gave them pretty much whatever you can think of, we gave them. We had big functions, like free big barbecues in the park, interactive with pageants. Anything that we could think of to get the people to come, because once you get them to come, 9 times out of 10 and somebody is gonna walk away with the vaccination”
“Then you got the food, you got the entertainment, you got free food. Then you got us on the side passing out pamphlets. Then you got a pharmacist that would get up and talk, maybe just a good 5 min. You do not want to be up too long boring the people. But just getting the point across, and just letting them know the importance of getting vaccinated”
“Yes. (It was important having the pharmacists in the vaccine education events) It’s kind of like going to the doctor’s office really. So, you go to the doctor’s office, and you have a doctor that comes in – sometimes a doctor does not even come in. You cannot ask them the questions you want, even though the nurse may know the answer. But you feel more comfortable when you have a one-on-one session with the pharmacist. Yeah, and pharmacists, they know medicine”
“I (CHW) went with 2 of the pharmacists, and both of them talked before we started giving the shots and they both did an excellent job, and to me, I think if they had not talked the way they talked, people would not have gotten their shots. I think they really rely on the pharmacists to know what they are doing and what they are saying, even though they did not even know them. Because I’m not talking about here in our town, I’m talking about we went away from our town to Marston, and we went to Haiti Heights. They listened, and they [the pharmacists] got the questions and they did a very good job”
“We had some negatives, but most of the people who came (to the outreach event/clinics) were at least interested… With the people I met (in-person) with at least we could have debates… a lot of the phone calls I made, people were like not interested, do not call me back”

**Table 4:** Trust was the key factor.

“Because I (CHW) already had a relationship with them (community members), and they trusted me, it seemed to be a lot easier for me to go in and talk to them and share education with them. They seem to take it a little bit better than what they were seeing on the news”
“Exactly trust, they (community people) already trusted me (CHW). Like I said already knowing who you are, and just being reliable person, trustworthy, because just because you know a person do not mean you trust, them”
“I (CHW) think we affect change because a lot of people trust us. Because we are so embedded in our communities”
There were still people who were anti vax, and I felt like they were more comfortable having the conversation with us because they knew us”
“It (vaccination) was just like a domino effect. If you can get one person to do it in front of the other one, you got them all lined up. They trust each other. And that’s what I’m saying is all about one little word and its trust”
“They (community people) got on Facebook saying, “We’ve been vaccinated, we have been vaccinated.” It (influenced peers to get vaccinated / look up information on vaccines) did, it was like a chain reaction”

## Discussion

This paper provides insight into the functioning of CHWs within an initiative implemented in rural Missouri through a community-academic partnership (10). The central role of CHWs in conducting various outreach events facilitated collaboration among local partner organizations, pharmacies, and community leaders. This further allowed their impact to go beyond providing information to the community members and increasing vaccine uptake. The efficacy of face-to-face conversation was a reoccurring theme among the CHWs. Apart from making people aware of facts about COVID-19 and providing them with authentic sources of information, the CHWs were also able to empower communities to fact-check information for themselves as well as connect people to resources such as transportation.

Our findings also indicate that CHWs have the potential to contribute to the health seeking behavior of their communities. Health seeking behavior includes recognizing that there is a concern significant enough to require seeking an appropriate remedy for it (11). However, evidence suggests that care seeking is a complex process influenced by many factors preceding the actual act of decision-making (12), and requiring contextual analysis. Social determinants of health often act as barriers and enablers to treatment decision making and health outcomes. CHWs that were a part of this community academic network were more equipped at understanding these contextual barriers as they were a part of the communities they were serving, and more importantly had their trust. This allowed them to address their health-related needs and placed them in a position to create structural and systems changes to begin to address associated social determinants. As noted by the CHWs, when engaging communities, health workers need to be aware of the social and political context within which this decision-making occurs. When governmental entities, public health, clinical and social services have acted in ways that indicate that they have not had the communities’ interests in mind, communities lose trust in these institutions and will view any new health requests, mandates, and suggestions through this lens of mistrust (13).

Our study strongly indicated that rural communities viewed COVID-19 messages and data through a contextual lens. CHWs were able to bridge what was often a lack of trust and dismantle the barriers because they were a part of the communities they served, they established relationships with the community, and they developed unique ways to engage with communities using locally appropriate strategies. This suggests that we need to consider the messages we provide, the way we provide them, who is providing the message and the current and historical relationships that the individual and/or community has with providers and the organizations they represent. Study findings strongly recommend that community health actions draw on the capacities of trusted members of the community for planning and implementing future educational interventions.

There is already a substantial body of evidence demonstrating the disparate burden of the adverse impact of COVID-19 on underserved and under-resourced communities (14, 15). In terms of process of engaging with such communities, the CHWs highlighted the need to meet people where they were and provide information in multiple ways and at multiple times; taking time to realize that health education needs to be a *process* of engagement and building trust, recognizing the historical and cultural perspectives of communities, not just telling people what to do. Although, CHWs saw the negative impact of social media on vaccine uptake, quoting it to be their ‘worst enemy’, in considering what information to provide, the CWHs emphasized the importance of providing community members with reliable and credible sources of information, in person and online, in ways that facilitated dialog and encouraged reflection and questions.

This is consistent with previous work that has noted that individuals who had questions about COVID-19 vaccines and used the internet as their main source of information about vaccines were less likely to obtain the vaccine (16). Some have suggested that this may be associated with the fact that search engines are designed to reflect individuals’ patterns of inquiry, thus if one finds one source that is critical of the vaccine, their future searches are likely to bring them to similarly framed sources. In this way the internet may actually be narrowing rather than broadening access to information (17). CHWs found that the best way to counter the negative impacts of these websites was to encourage people to begin to confirm information for themselves, providing them with online news sources and authentic websites that might help expand their views (and their future searches), rather than just deepen one perspective. As health educators, we need to recognize and encourage the agency of communities in improving their own health and the need to create the conditions within which they can do so. We need to recognize the potential for benefit from technology but temper it with providing it with both strategies for vetting information on the web and interpersonal interactions.

The CHWs also identified that having CHWs working with and across agencies enhanced coordination among agencies and enhanced the ways that agencies were able to listen to community needs, opening the door to addressing social determinants of health, through lasting partnerships, not just providing direct care.

### Limitations

Study findings are consistent with previous work in the field that suggests that vaccine uptake is influenced by the degree to which individuals understand the reasons for obtaining a vaccine, agree to getting the vaccine, and are moved toward obtaining a vaccine (awareness, acceptance, and activation) (18). However, within the context of our project, COVID-19 vaccines were brought to community sites, where people were otherwise gathered, and were offered for free. This reduces the generalizability of our findings, given that they do not highlight two other critical determinants of vaccine uptake, affordability and accessibility.

## Conclusion

CHWs are a vital health care resource within our communities. Their knowledge, experiences, and pre-established relationships with community members facilitate program implementation and improve health outcomes in the community. Within rural communities, where healthcare systems are often inadequate, our work suggests that CHWs can also contribute to collaboration among community organizations, social service agencies, and health care providers (including pharmacies) and enhance their ability to positively impact health outcomes.

## Data availability statement

Aggregate, deidentified data will be made available, without undue reservation. However raw qualitative data cannot be provided without compromising the confidentiality of our participants. Further inquiries can be directed to the corresponding author.

## Ethics statement

The studies involving humans were approved by Saint Louis University Institutional Review Board. The studies were conducted in accordance with the local legislation and institutional requirements. Given the nature of the study, the IRB determined that written consent was not necessary and instead participants provided verbal informed consent to participate in this study and for the publication of aggregate, deidentified data.

## Author contributions

AB: Conceptualization, Data curation, Investigation, Methodology, Writing – original draft, Project administration. AS: Writing – review & editing, Data curation. CV: Conceptualization, Funding acquisition, Investigation, Project administration, Supervision, Writing – review & editing. JB: Conceptualization, Funding acquisition, Project administration, Writing – review & editing. RL: Project administration, Resources, Writing – review & editing, Data curation. TL: Project administration, Resources, Writing – review & editing, Data curation. AF: Project administration, Project administration, Data curation. MM: Project administration, Resources, Writing – review & editing. EB: Conceptualization, Funding acquisition, Investigation, Project administration, Resources, Supervision, Writing – original draft, Data curation, Formal analysis.
