# Supplementary material for: Through the eyes of community health workers: what was needed to increase COVID-19 vaccine uptake in the Missouri Southeast region
Source: Front Public Health. 2024 Mar 27;12:1286177. doi: 10.3389/fpubh.2024.1286177 (PMC11004485; doi:10.3389/fpubh.2024.1286177)
Supplement: Supplementary file 1 [file Table_1.DOCX]

Table of Contents

[Content Description 2](#_Toc159837325)

[CHW Interview Protocol 2](#_Toc159837326)

# **Content Description**

This supplemental file consists of the interview protocol used to collect data from the study participants.

# **CHW Interview Protocol**

We are currently reaching out to past and current Network participants for input that can help us continue to build the network going forward. We’d like to hear your thoughts on what is going well, what we can do differently, and what our future work might look like. Your perspective is crucial to the network's growth and continuity.

If you can remember, **what was your perspective about the pandemic** before you started working on this project?  In **what ways did this change** since you’ve been working on the project?

Next, we’d like to know about **your experiences** being a community health worker for this COVID-19 project:

- How did you find out about getting the **opportunity to be a CHW** on this project?
- How would you describe **your role as a CHW** as part of the COVID19 project?
- How did the **community respond to you** in this role?
- Overall, what has been **your experience working with the community** to provide information about COVID -19 and COVID-19 vaccinations?
  - Has it been **positive or negative**?  How so?

We are interested in some of **what you saw and heard** while you were working in the community.  Can you tell us **some examples** about your interactions with community members?

- Around **COVID-19 education**?
- Around the **COVID-19 vaccine**?

What were some of the **strategies you used to talk with people** about COVID-19 in your community?

- Which ones did you feel were the **most effective**?
- Which ones just **did not work**?

Now we’d like to ask you some questions about training.

- What is **your opinion of the training you received** (with Megan Murray, Brooke Clubbs)?
- In what ways did these **trainings prepare you** for this work?
- What could have been **done differently**?  What else would you have liked **information on**?

We also tried to create some other types of support.  In general, **did you have the support you needed** to engage in this work?

- Prompts:
  - What about the **reflective supervision** meetings with **Shelley Mayse**?
  - What about the **additional practice sessions/problem solving** with **Brooke Clubbs**?
  - What about **meetings among CHWs**- **how useful** were they?  Was the Frequency appropriate?  What about the **content**?
  - **Other things** you might have appreciated?
  - What could you have **done without**?
  - What are some of the things **you needed but were not available**?

In what ways were you able to help to **create changes in health care/public health** or **other systems and structures** as a result of your role on the project (for example, do you think you were able to make **any changes within the organizations where you work** or **agencies you interact with**?  What about **referral systems between agencies**? Any changes in things like **transportation, access to testing, contract tracing**?

Our next set of questions have to do with **CHW personal and professional development**.

- In what ways, if any, did your work on this project help **your career or job prospects**?
- In what ways, if any, did your work on this project help **your personal development** (i.e., how you feel about **yourself, relationships, knowledge about health,** etc.)?
- In what ways did this COVID-19 work **help or take away from how you  are viewed at your job?** [if applicable – Logans, WKO, OHMH if part of another agency]

We now have a question about CHWs more generally.

- In general, in what ways are **CHWs an important resource in your community**?

***
